# Changes in Dietary Habits and Exercise Pattern of Korean Adolescents from Prior to during the COVID-19 Pandemic

**DOI:** 10.3390/nu13103314

**Published:** 2021-09-23

**Authors:** So Young Kim, Dae Myoung Yoo, Chanyang Min, Hyo Geun Choi

**Affiliations:** 1 Department of Otorhinolaryngology-Head & Neck Surgery, CHA Bundang Medical Center, CHA University, Seongnam 13496, Korea; sossi81@hanmail.net; 2 Hallym Data Science Laboratory, Hallym University College of Medicine, Anyang 14068, Korea; ydm1285@naver.com (D.M.Y.); joicemin@naver.com (C.M.); 3 Graduate School of Public Health, Seoul National University, Seoul 08826, Korea; 4 Department of Otorhinolaryngology-Head & Neck Surgery, Hallym University College of Medicine, Anyang 14068, Korea

**Keywords:** COVID-19, dietary habits, exercise pattern, adolescents, cohort study

## Abstract

This study aimed to investigate changes in the exercise pattern and dietary habits in adolescents during the COVID-19 pandemic. The 12–18-year-old population in the Korea Youth Risk Behavior Web-Based Survey data of 2019 and 2020 was enrolled. The exercise pattern and dietary habits of 105,600 participants (53,461 in the 2019 group and 52,139 in the 2020 group) were compared. The odds ratios (ORs) for the dietary habits and exercise pattern of the 2020 group compared to the 2019 group were analyzed using multiple logistic regression analysis with complex sampling. The odds of eating fruit, drinking soda, drinking sweet drinks, and consuming fast food were lower in the 2020 group than in the 2019 group (all *p* < 0.001). The odds of eating breakfast were higher in the 2020 group than in the 2019 group (all *p* < 0.001). The 2020 group showed lower odds of frequent vigorous and moderate aerobic exercise and higher odds of frequent anaerobic exercise than the 2019 group (all *p* < 0.001). During the COVID-19 pandemic, adolescents consumed less fruit, soda, and sweet drinks, while they had more breakfast. The frequency of aerobic exercise was lower, while the frequency of anaerobic exercise were higher during the COVID-19 pandemic period.

## 1. Introduction

The outbreak of COVID-19 had various impacts on our daily lives [1]. Due to the high infectivity of SARS-CoV-2, the prevention of contagious infection and the isolation of COVID-19 patients have been among the most important strategies in response to the COVID-19 pandemic. The quarantine maneuvers included social distancing, restriction of social activities, and lockdown. These limitations on social circumferences increased the time spent indoors and eating home-cooked meals, while they decreased outdoor activities and eating out [2]. The lockdown of schools and workplaces changed the schedules of daily life for many people.

A number of researchers have been concerned about the changes in dietary habits and exercise pattern during the COVID-19 pandemic [3,4,5,6,7]. A cross-sectional survey in Poland found that approximately 43% and 52% of adults consumed more food and snacks, respectively, during the COVID-19 lockdown [3]. Approximately 30% of participants gained weight because of the frequent consumption of fast food (3.0 ± 1.6 kg) during the COVID-19 lockdown [3]. Another survey in Italy reported that approximately 33.5% of participants changed their eating habits, and approximately 81% of participants increased frozen food consumption during the COVID-19 lockdown [5]. This survey estimated that home confinement during the COVID-19 crisis was negatively associated with physical activity intensity and positively associated with sedentary time during the COVID-19 lockdown [5].

Importantly, adolescents are in periods of development and are susceptible to acquiring poor lifestyle patterns, including habits related to diet and exercise pattern [5,8,9]. Indeed, an international online survey reported that adolescents consumed more fast food and sweet food during home confinement due to the COVID-19 lockdown (44.6% vs. 64% for fast food; 14% vs. 20.7% for sweet food) [5]. In addition, 70.5% of participants reduced their exercise pattern during the COVID-19 lockdown [5]. An international online survey demonstrated reduced physical activity levels (vigorous, moderate, walking, and overall) during home confinement due to the COVID-19 crisis (5.04 ± 2.51 day/week vs. 3.83 ± 2.82 day/week of physical activities, *p* < 0.001) [6]. However, the information on the respondents was very limited, and the analysis did not consider possible confounders, such as economic status, sleep time, and obesity. As multiple lifestyle factors influence dietary habits and exercise pattern, these factors should be concurrently considered in analyses of the impact of the COVID-19 pandemic on diet and exercise pattern.

As changes in dietary habits and exercise pattern during the COVID-19 pandemic were suggested to increase the risk of metabolic disorders in a previous study [10], we hypothesized that there may have also been changes in the dietary habits and physical activity during the COVID-19 pandemic in Korean adolescents. To test this hypothesis, the dietary habits and exercise pattern were analyzed in adolescents in before and during the COVID-19 pandemic periods. The first patient with COVID-19 was diagnosed on 19 February 2020. Thus, this study compared the 2019 group as a prepandemic population (surveyed from 3 June 2019 to 12 July 2019) with the 2020 group as a COVID-19 pandemic population (surveyed from 3 August 2020 to 13 November 2020 during the social distancing strategies that had been maintained in Korea without complete lockdowns).

## 2. Materials and Methods

### 2.1. Study Population and Data Collection

This cross-sectional study used data from the Korea Youth Risk Behavior Web-based Survey (KYRBWS) and covered the nation using statistical methods based on the designed sampling and adjusted weighted values. The KYRBWS obtained data from South Korean adolescents using stratified, two-stage (schools and classes) clustered sampling based on data from the Education Ministry. Sampling was weighted by statisticians, who performed poststratification analyses and considered the nonresponse rates and extreme values. Data from the 2019 and 2020 KYRBWS were analyzed. The details of the sampling methods are described on the KYRBWS website [11]. The KCDC collected the data, and Korean adolescents from 7th through 12th grade voluntarily and anonymously completed the self-administered questionnaire. The validity and reliability of the KYRBWS have been documented by other studies [12,13].

**Figure 1:**
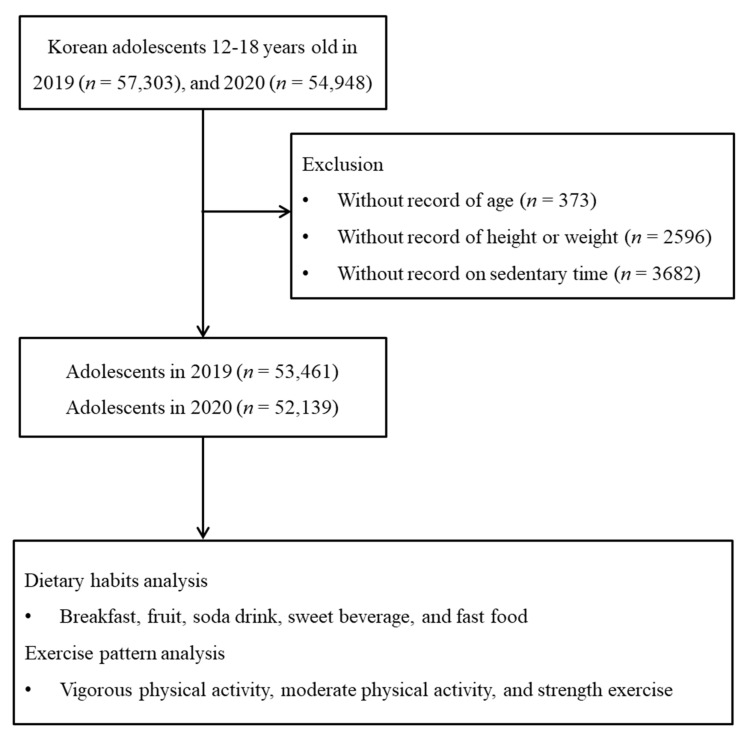
Flowchart of the major steps involved in the conduction of this study.

Of the 112,251 total participants (57,303 in 2019; 54,948 in 2020), the following were excluded from this study: participants without information on age (*n* = 373), height or weight (*n* = 2596), and sedentary time (*n* = 3682). Finally, 105,600 participants (53,461 in 2019; 52,139 in 2020), who were 12 through 18 years old, were included in this study (Figure 1).

### 2.2. Survey

#### 2.2.1. Exposure

In each of the 2019 and 2020 surveys, adolescent participants were selected as stated above to represent the entire adolescent population in Korea. The participants from 2019 were not followed up with. The participants of 2020 were newly selected from the entire Korean adolescent population.

#### 2.2.2. Outcomes

Dietary habits were surveyed by asking about the frequency of particular dietary habits in the last week. The frequencies of breakfast, fresh fruit, soda beverage (except for pure soda drinks and caffeinated drinks), sweet drinks, and fast food intake were assessed using questionnares. “How many days did you eat breakfast, fresh fruit, soda beverage (except for pure soda drinks and caffeinated drinks), sweet drink, and fast food in the recent 7 days, respectively?”.

Regarding exercise pattern, aerobic exercise involving vigorous physical activity was assessed with the following question: “How many days did you exercise with high intense enough to sweat more than 20 min in the recent 7 days?”. Aerobic exercise involving moderate physical activity was assessed by asking: “How many days did you exercise until heart rate increase or be short of breath more than 60 min in the recent 7 days?”. Anaerobic strength exercises were assessed by asking: “How many days did you exercise to increase muscle power such as sit-up, lift weight, or chin-up bar in the recent 7 days?”.

#### 2.2.3. Covariates

Body mass index (BMI, kg/m^2^) was calculated using height and weight. Mean sedentary time (h/day) and leisure time were calculated as 5/7 of the time on weekdays plus 2/7 of the time on weekends [14,15]. Sleep times were calculated as 5/7 of the time on weekdays plus 2/7 of the time on weekends [14,15]. The self-reported economic level was categorized into 3 levels: high, middle, and low. Subjective self-reported health status was categorized into 4 levels that ranged from very healthy to unhealthy. Subjective body shape image was categorized into 3 levels, including thin, normal, and obese. Smoking in the last 30 days was assessed and categorized into 3 levels: 0, 1–19, and ≥20 days [16]. Drinking alcohol in the last 30 days was categorized into 3 levels: 0, 1–2, and ≥3 days [16].

### 2.3. Statistical Analysis

The general characteristics of the data from the 2019 and 2020 surveys were compared using *t*-tests and chi-square tests.

The odds ratios (ORs) for particular dietary habits (breakfast, fresh fruit consumption, soda beverage, sweet drinks, and fast food intake) and exercise pattern (vigorous physical activity, moderate physical activity, and strength exercise) from 2020 compared to 2019 were calculated using multiple logistic regression analysis with complex sampling.

Crude and partially adjusted (age, BMI, sedentary time for study or leisure, sex, economic level, sleep time, subjective health status, subjective body shape image, and smoking and alcohol consumption) and fully adjusted (partial model plus dietary habits and exercise pattern) models were designed. Subgroups determined by sex and school level (middle school vs. high school) were analyzed.

Two-tailed analyses were conducted, and *p*-values lower than 0.05 were considered to indicate significance; 95% confidence intervals (Cis) were also calculated. The weights recommended by the KYRBWS were applied, and thus complex sampling was applied. The data were analyzed using SPSS ver. 25.0 (IBM, Armonk, NY, USA).

### 2.4. Ethics Approval

The ethics committee of Hallym University approved the use of these data. The study was exempted from the need for written informed consent by the Institutional Review Board (2019-09-005). All Korea Youth Risk Behavior Web-Based Survey (KYRBWS) data analyses were conducted in accordance with the guidelines and regulations provided by the Institutional Review Board of the Centers for Disease Control and Prevention of Korea (KCDC). The understanding, reliability, and validity of each question were investigated by the KCDC to verify the applicability of the surveys [11].

## 3. Results

The average sedentary time reported in the study was 6.6 (±3.7) hours/day in the 2019 group and 5.9 (±3.4) hours/day in the 2020 group (*p* < 0.001; Table 1). The average sedentary time for leisure was 3.3 (±2.3) hours/day in the 2019 group and 4.3 (±2.8) hours/day in the 2020 group (*p* < 0.001). Reporting subjective body shape image as obese was lower in the 2019 group than in the 2020 group (37.5% vs. 38.5%, *p* < 0.001). The frequencies of smoking and alcohol consumption were higher in the 2019 group than in the 2020 group (*p* < 0.001). The distributions of BMI, sleep time, and subjective health status were different between the 2019 group and the 2020 group (all *p* < 0.001).

In the fully adjusted model, the 2020 group demonstrated lower odds of eating fruit, fast food, drinking soda, and drinking sweet drinks than the 2019 group (all *p* < 0.001; Table 2). On the other hand, the frequency of breakfast was lower in the 2019 group than in the 2020 group (*p* < 0.001). In middle school students, the frequencies of eating fruit, drinking soda, drinking sweet drinks, and eating fast food were lower in the 2020 group than in the 2019 group (all *p* < 0.001; Table S1). In high school students, the frequencies of eating fruit, drinking soda, and drinking sweet drinks were lower in the 2020 group than in the 2019 group (all *p* < 0.001, Table S2). When analyzed by sex, men showed lower frequencies of drinking soda and sweet drinks in the 2020 group than in the 2019 group (all *p* < 0.001; Table S3). In women, the frequencies of eating fruit, drinking sweet drinks, and eating fast food were lower in the 2020 group than in the 2019 group (all *p* < 0.05, Table S4).

Regarding exercise pattern, the frequencies of vigorous and moderate aerobic exercise were lower in the 2020 group than in the 2019 group (all *p* < 0.001; Table 3). The 2020 group showed lower odds regarding the frequency of vigorous and moderate aerobic exercise than the 2019 group (all *p* < 0.001). In contrast, the frequency of anaerobic exercise was higher in the 2020 group than in the 2019 group (*p* < 0.001). The 2020 group showed higher odds regarding the frequency of anerobic exercise than the 2019 group (*p* < 0.001). Middle school students showed a lower frequency of vigorous aerobic exercise and a higher frequency of anaerobic exercise in the 2020 group than in the 2019 group (all *p* < 0.001; Table S1). High school students showed lower frequencies of vigorous and moderate aerobic exercise and a higher frequency of anaerobic exercise in the 2020 group than in the 2019 group (all *p* < 0.001; Table S2). When analyzed by sex, both men and women showed a lower frequency of vigorous aerobic exercise and a higher frequency of anaerobic exercise in the 2020 group than in the 2019 group (all *p* < 0.001; Tables S3 and S4).

## 4. Discussion

The consumption of fast food, fruit, and drinks such as soda was lower, while the consumption of breakfast was higher during the COVID-19 crisis than in the prepandemic era. The pattern of exercise showed bidirectional changes, with lower aerobic exercise and higher anaerobic exercise during the COVID-19 pandemic when compared with the prepandemic period in this cohort of Korean adolescents. This study improved previous findings on the impact of the COVID-19 pandemic on dietary habits and exercise pattern by concurrently analyzing both the dietary habits and exercise pattern in an adolescent population. In addition, age and sex were subgrouped, and the impact of the COVID-19 pandemic on diet and exercise pattern was analyzed in each subgroup.

The COVID-19 pandemic was associated with decreased intakes of fast food, fruit, soda, and sweet drinks in this cohort of Koreran adolescents. On the other hand, the frequency of breakfast was increased during the COVID-19 pandemic. The decreased consumption of soda, sweet drinks, and fast food could be explained by less frequent eating out due to home confinement. The consumption of fast food and soda intake have mostly been associated with eating out in Korean adolescents [17]. In addition, health-seeking behavior could have decreased the consumption of unhealthy food during the COVID-19 pandemic in this study. The COVID-19 pandemic could have encouraged adolescents to address health-related issues because they were repeatedly exposed to health campaigns and education regarding quarantine maneuvers from school and the mass media. Moreover, the increased time with family and frequency of home cooking could have provided an opportunity for adolescents to discuss healthy behavior with their parents and to alter their unhealthy dietary habits [18,19]. The traditional Korean diet at home includes cooked rice and a number of banchan, such as kimchi. Thus, Koreans could consume sufficient nutrients from vegetables, legumes, and fish, with a low intake of red meat [20]. Frequently cooking dinner was related to a higher Healthy Eating Index-2015 in the US (≥7 times/week: +3.57 points, *p* < 0.001) [19]. During the COVID-19 crisis, a cross-sectional survey in Italy demonstrated that approximately 3.3% of respondents quit smoking and 38.3% of respondents increased physical activities, such as bodyweight training [4]. The lockdown and other instances of quarantine could have resulted in difficulties acquiring food, especially fresh food, such as fruit, in some adolescents without caregivers. Indeed, it was reported that over 18% of adults lost weight during the COVID-19 lockdown (−2.9 ± 1.5 kg) [3]. In our cohort, although the BMI was slightly higher in the COVID-19 pandemic group than in the prepandemic group, it was not high enough to have a clinical impact. On the other hand, the increasd time to eat due to the lockdown of schools could have resulted in increasing breakfast consumption, which was observed in this study. Because the restriction of outdoor activities can increase the time available to eat, the increase in breakfast consumption could have been relative to the irregular meal time during school hours.

Anaerobic exercise was higher in the COVID-19 pandemic group than in the prepandemic group in this study. In contrast, vigorous aerobic exercise was lower in the COVID-19 pandemic group than in the prepandemic group. A previous survey estimated 1.3-fold and 1.2-fold higher rates of moderate-to-vigorous physical activity in male and middle-aged populations during the COVID-19 pandemic [21]. The increase in anaerobic exercise could have been a compensation for the decrease in aerobic exercise during the COVID-19 pandemic that was found in the study. A cross-sectional study reported that children spent less time in extracurricular sports (23.5%), and 94.5% of children watched screens for 1.5 (0.5–3.0) hours/day [22]. Quarantine and social distancing maneuvers might have limited outdoor activities [23]. The restrictions on physical activities during the COVID-19 pandemic may have been caused by both individual and community aspects [24,25]. Physical restrictions on outdoor activities may have reduced individuals’ aerobic exercise. In addition, the social distancing policy included restrictions on group sports activities, such as football leagues, swimming, and baseball games, all of which are forms of aerobic exercise. On the other hand, the increased time spent at home may have encouraged people to spend time at home training. Moreover, awareness of health-related issues via health promotion campaigns encouraged people to engage in home-based exercises [23,26]. Indeed, a surveillance in Thailand reported a 1.5-fold higher rate of moderate-to-vigorous physical activity in adults who were exposed to fit-from-home campaigns [21]. Furthermore, physical activity was reported to be greatly influenced by personal factors in children, while sedentary behavior was influenced by environmental factors [27]. Thus, physical activity was less influenced by the COVID-19 pandemic, but the geographic area of activity decreased.

The present cohort was composed of a large, nationwide, representative population of Korean adolescents. The validation and regulation of the data were managed by KCDC. Many surveyed items of sedentary time for study, sedentary time for leisure, economic level, sleep time, subjective health status, subjective body shape image, smoking, alcohol consumption, and anthropometric index of BMI were included for analyses. We included these variables as they might have been able to affect diet and exercise pattern. However, because this study was based on the survey, recall bias may have existed. In addition, the amount of food consumption could not be evaluated. The detailed types of physical exercise could not be identified. Furthermore, the study participants could not be longitudinally followed up with; instead, new participants were enrolled in each year. Although numerous factors were surveyed and adjusted for, influential variables may have been unmeasured, such as comorbidities that restrict dietary consumption or exercise pattern. Lastly, because this study included a Korean population, ethnic or regional differences could be present regarding dietary habits or exercise pattern of other ethnic groups [28].

## 5. Conclusions

There were changes in both the dietary habits and pattern of exercise between the pre- and during COVID-19 pandemic periods in this cohort of Korean adolescents. The consumption of fruit, fast food, soda, and sweet drinks was lower, while the consumption of breakfast was higher during the COVID-19 pandemic than in the prepandemic period. The frequencies of vigorous and moderate aerobic exercise were lower, while the frequency of anerobic exercise was higher during the COVID-19 pandemic than in the prepandemic period.

## Figures and Tables

**Table 1:** General Characteristics of Participants.

General Characteristics		Participated Year		
		2019	2020	*p*-Value
Total Number, *n* (%)		53,461 (100.0)	52,139 (100.0)	
Age (years, mean (SD))		15.0 (1.8)	15.1 (1.8)	<0.001 *
BMI (kg/m^2^, mean (SD))		21.3 (3.6)	21.5 (3.7)	<0.001 *
Sedentary time for study (hour/day, mean (SD))		6.6 (3.7)	5.9 (3.4)	<0.001 *
Sedentary time for leisure (hour/day, mean (SD))		3.3 (2.3)	4.3 (2.8)	<0.001 *
Sex, *n* (%)				0.726
	Male	27,776 (52.0)	27,033 (51.8)	
	Female	25,685 (48.0)	25,106 (48.2)	
Economic level, *n* (%*)				0.382
	High	20,979 (39.2)	20,367 (39.1)	
	Middle	25,767 (48.2)	25,076 (48.1)	
	Low	6715 (12.6)	6696 (12.8)	
Sleep time, *n* (%*)				<0.001 †
	Unknown or missing	5018 (9.4)	7923 (15.2)	
	< 6 h	12,370 (23.1)	11,911 (22.8)	
	6 h to <7 h	12,155 (22.7)	11,440 (21.9)	
	7 h to <8 h	11,548 (21.6)	10,375 (19.9)	
	≥8 h	12,370 (23.1)	10,490 (20.1)	
Subjective health status, *n* (%*)				0.007 †
	Very healthy	14,451 (27.0)	14,447 (27.7)	
	Healthy	23,383 (43.7)	22,276 (42.7)	
	Normal	11,848 (22.2)	11,634 (22.3)	
	Unhealthy	3779 (7.1)	3782 (7.3)	
Subjective body shape image, *n* (%*)				<0.001 †
	Thin	13,768 (25.8)	12,820 (24.6)	
	Normal	19,662 (36.8)	19,220 (36.9)	
	Obese	20,031 (37.5)	20,099 (38.5)	
Smoking in the recent 30 days, *n* (%*)				<0.001 †
	0 day	50,205 (93.9)	49,886 (95.7)	
	1–19 days	1473 (2.8)	871 (1.7)	
	≥20 days	1783 (3.3)	1382 (2.7)	
Drinking alcohol in the recent 30 days, *n* (%*)				<0.001 †
	0 day	45,879 (85.8)	46,679 (89.5)	
	1–2 days	4525 (8.5)	3300 (6.3)	
	≥3 days	3057 (5.7)	2160 (4.1)	

* Independent *t*-test, Significance at *p* < 0.05. † Chi-square test, Significance at *p* < 0.05.

**Table 2:** Odd ratios of dietary habits in 2020 compared to 2019 in total participants.

Dietary Habit		Number (%)		OR (95% CI)					
		2019	2020	Crude	*p*-Value	Partial †	*p*-Value	Full ‡	*p*-Value
Breakfast					<0.001 *		0.008 *		<0.001 *
	0–1 time/week	14,455 (27.0)	14,786 (28.4)	1.00 (Ref)		1.00 (Ref)		1.00 (Ref)	
	2–4 times/week	12,471 (23.3)	12,862 (24.7)	1.02 (0.98–1.05)		1.05 (1.01–1.09)		1.07 (1.03–1.11)	
	≥5 times/week	26,535 (49.6)	24,491 (47.0)	0.91 (0.87–0.95)		1.00 (0.96–1.03)		1.01 (0.97–1.05)	
Fruit					<0.001 *		<0.001 *		<0.001 *
	0–2 times/week	22,064 (41.3)	23,334 (44.8)	1.00 (Ref)		1.00 (Ref)		1.00 (Ref)	
	3–4 times/week	14,806 (27.7)	13,922 (26.7)	0.90 (0.87–0.93)		0.93 (0.90–0.97)		0.95 (0.92–0.99)	
	≥5 times/week	16,591 (31.0)	14,883 (28.5)	0.86 (0.82–0.89)		0.91 (0.88–0.95)		0.93 (0.89–0.96)	
Soda drink					<0.001 *		<0.001 *		<0.001 *
	0–2 times/week	33,928 (63.5)	33,667 (64.6)	1.00 (Ref)		1.00 (Ref)		1.00 (Ref)	
	3–4 times/week	12,533 (23.4)	11,482 (22.0)	0.92 (0.88–0.96)		0.87 (0.84–0.90)		0.92 (0.88–0.95)	
	≥5 times/week	7000 (13.1)	6990 (13.4)	0.99 (0.94–1.04)		0.89 (0.85–0.93)		0.94 (0.90–0.99)	
Sweet drinks					<0.001 *		<0.001 *		<0.001 *
	0–2 times/week	26,803 (50.1)	28,229 (54.1)	1.00 (Ref)		1.00 (Ref)		1.00 (Ref)	
	3–4 times/week	15,607 (29.2)	13,350 (25.6)	0.80 (0.77–0.82)		0.79 (0.76–0.81)		0.81 (0.78–0.84)	
	≥5 times/week	11,051 (20.7)	10,560 (20.3)	0.90 (0.86–0.93)		0.85 (0.82–0.89)		0.90 (0.86–0.94)	
Fast food					0.191		<0.001 *		0.001 *
	0–2 times/week	40,236 (75.3)	39,263 (75.3)	1.00 (Ref)		1.00 (Ref)		1.00 (Ref)	
	3–4 times/week	10,487 (19.6)	10,316 (19.8)	1.01 (0.98–1.05)		0.97 (0.93–1.00)		1.02 (0.98–1.05)	
	≥5 times/week	2738 (5.1)	2560 (4.9)	0.96 (0.90–1.02)		0.84 (0.79–0.89)		0.90 (0.85–0.96)	

* Multiple logistic regression analysis with complex sampling, Significance at *p* < 0.05; † Adjusted for age, BMI, sedentary time for study or leisure, sex, economic level, sleep time, subjective health status, subjective body shape image, smoking, and drinking alcohol histories. ‡ Adjusted for partial model plus dietary habit and exercise pattern.

**Table 3:** Odd ratios of exercise pattern in 2020 compared to 2019 in total participants.

Physical Activity (PA)		Number (%)		OR (95% CI)					
		2019	2020	Crude	*p*-Value	Partial †	*p*-Value	Full ‡	*p*-Value
Vigorous PA					<0.001 *		<0.001 *		<0.001 *
	0 time/week	16,819 (31.5)	19,859 (38.1)	1.00 (Ref)		1.00 (Ref)		1.00 (Ref)	
	1–2 times/week	19,123 (35.8)	17,411 (33.4)	0.76 (0.73–0.81)		0.78 (0.74–0.81)		0.74 (0.71–0.77)	
	≥3 times/week	17,519 (32.8)	14,869 (28.5)	0.71 (0.67–0.76)		0.71 (0.68–0.75)		0.60 (0.57–0.63)	
Moderate PA					<0.001 *		<0.001 *		0.719
	0 time/week	18,647 (34.9)	20,008 (38.4)	1.00 (Ref)		1.00 (Ref)		1.00 (Ref)	
	1–2 times/week	16,210 (30.3)	15,317 (29.4)	0.88 (0.84–0.92)		0.91 (0.87–0.94)		0.99 (0.95–1.02)	
	≥3 times/week	18,604 (34.8)	16,814 (32.2)	0.84 (0.79–0.89)		0.87 (0.83–0.91)		0.99 (0.96–1.04)	
Strength Exercise					<0.001 *		<0.001 *		<0.001 *
	0 time/week	27,721 (51.9)	26,074 (50.0)	1.00 (Ref)		1.00 (Ref)		1.00 (Ref)	
	1–2 times/week	13,912 (26.0)	13,124 (25.2)	1.00 (0.95–1.05)		1.05 (1.01–1.10)		1.19 (1.14–1.24)	
	≥3 times/week	11,828 (22.1)	12,941 (24.8)	1.15 (1.07–1.23)		1.23 (1.17–1.28)		1.54 (1.46–1.62)	

* Multiple logistic regression analysis with complex sampling, Significance at *p* < 0.05. † Adjusted for age, BMI, sedentary time for study or leisure, sex, economic level, sleep time, subjective health status, subjective body shape image, smoking, and drinking alcohol histories. ‡ Adjusted for partial model plus dietary habit and physical activities.

## Data Availability

Releasing of the data by the researcher is not legally permitted. All data are available from the database of the Korea Center for Disease Control and Prevention. The Korea Center for Disease Control and Prevention allows data access, at a particular cost, for any researcher who promises to follow the research ethics. The data of this article can be downloaded from the website after agreeing to follow the research ethics.

## Supplementary Materials

The following are available online at https://www.mdpi.com/article/10.3390/nu13103314/s1, Table S1. Odd ratios of dietary habit or exercise pattern in 2020 compared to 2019 in middle school student. Table S2. Odd ratios of dietary habit or exercise pattern in 2020 compared to 2019 in high school student. Table S3. Odd ratios of dietary habit or exercise pattern in 2020 compared to 2019 in men. Table S4. Odd ratios of dietary habit or physical activities in 2020 compared to 2019 in women.
