# Supplementary material for: Changes in Dietary Habits and Exercise Pattern of Korean Adolescents from Prior to during the COVID-19 Pandemic
Source: Nutrients. 2021 Sep 23;13(10):3314. doi: 10.3390/nu13103314 (PMC8541287; doi:10.3390/nu13103314)
Supplement: Supplementary file 1 [file nutrients-13-03314-s001.zip › nutrients-1365770-supplementary.pdf]

S1 Table Odd ratios of dietary habit or exercise pattern in 2020 compared to 2019 in middle school student.

Dietary habit or PA	Number (%)		OR (95% CI)					
	2019	2020	Crude	P-value	Partial†	P-value	Full‡	P-value
Breakfast				<0.001*		<0.001*		<0.001*
0-1 time/week	7,376 (26.9)	7,548 (27.4)	1.00 (Ref)		1.00 (Ref)		1.00 (Ref)	
2-4 times/week	6,140 (22.4)	6,758 (24.5)	1.10 (1.04-1.15)		1.28 (1.07-1.19)		1.16 (1.10-1.22)	
≥ 5 times/week	13,938 (50.8)	13,234 (48.1)	0.94 (0.90-0.99)		1.05 (1.00-1.11)		1.07 (1.02-1.13)	
Fruit				<0.001*		<0.001*		<0.001*
0-2 times/week	10,255 (37.4)	11,373 (41.3)	1.00 (Ref)		1.00 (Ref)		1.00 (Ref)	
3-4 times/week	7,520 (27.4)	7,369 (26.8)	0.88 (0.84-0.93)		0.94 (0.89-0.98)		0.95 (0.91-1.00)	
≥ 5 times/week	9,679 (35.3)	8,798 (31.9)	0.81 (0.77-0.86)		0.90 (0.85-0.95)		0.92 (0.87-0.97)	
Soda drink				<0.001*		<0.001*		<0.001*
0-2 times/week	17,455 (63.6)	18,003 (65.4)	1.00 (Ref)		1.00 (Ref)		1.00 (Ref)	
3-4 times/week	6,324 (23.0)	5,834 (21.2)	0.89 (0.84-0.94)		0.82 (0.78-0.86)		0.90 (0.86-0.95)	
≥ 5 times/week	3,675 (13.4)	3,703 (13.4)	0.97 (0.90-1.04)		0.85 (0.80-0.91)		0.96 (0.89-1.02)	
Sweet drink				<0.001*		<0.001*		<0.001*
0-2 times/week	14,366 (52.3)	15,829 (57.5)	1.00 (Ref)		1.00 (Ref)		1.00 (Ref)	
3-4 times/week	7,814 (28.5)	6,690 (24.3)	0.76 (0.73-0.80)		0.74 (0.70-0.77)		0.77 (0.74-0.81)	
≥ 5 times/week	5,274 (19.2)	5,021 (18.2)	0.85 (0.80-0.90)		0.77 (0.73-0.82)		0.84 (0.79-0.86)	
Fast food				0.004*		<0.001*		<0.001*
0-2 times/week	20,872 (76.0)	21,218 (77.0)	1.00 (Ref)		1.00 (Ref)		1.00 (Ref)	
3-4 times/week	5,171 (18.8)	5,026 (18.2)	0.94 (0.90-0.99)		0.87 (0.83-0.91)		0.92 (0.88-0.97)	
≥ 5 times/week	1,411 (5.1)	1,296 (4.7)	0.89 (0.82-0.97)		0.77 (0.71-0.83)		0.84 (0.78-0.92)	
Vigorous PA				<0.001*		<0.001*		<0.001*

0 time/week	6,866 (25.0)	9,174 (33.3)	1.00 (Ref)		1.00 (Ref)		1.00 (Ref)	
1-2 times/week	9,779 (35.6)	9,573 (34.8)	0.73 (0.69-0.78)		0.75 (0.71-0.80)		0.71 (0.68-0.75)	
≥ 3 times/week	10,809 (39.4)	8,793 (31.9)	0.62 (0.57-0.67)		0.63 (0.59-0.67)		0.54 (0.51-0.58)	
Moderate PA				<0.001*		<0.001*		0.105
0 time/week	8,613 (31.4)	9,657 (35.1)	1.00 (Ref)		1.00 (Ref)		1.00 (Ref)	
1-2 times/week	7,637 (27.8)	7,971 (28.9)	0.94 (0.89-0.99)		0.97 (0.92-1.02)		1.05 (1.00-1.11)	
≥ 3 times/week	11,204 (40.8)	9,912 (36.0)	0.80 (0.75-0.85)		0.84 (0.79-0.89)		1.01 (0.95-1.07)	
Strength Exercise				<0.001*		<0.001*		<0.001*
0 time/week	13,077 (47.6)	12,959 (47.1)	1.00 (Ref)		1.00 (Ref)		1.00 (Ref)	
1-2 times/week	8,048 (29.3)	7,681 (27.9)	0.97 (0.91-1.03)		1.04 (0.98-1.10)		1.19 (1.12-1.26)	
≥ 3 times/week	6,329 (23.1)	6,900 (25.1)	1.10 (1.01-1.18)		1.18 (1.11-1.26)		1.54 (1.44-1.65)	

* Multiple logistic regression analysis with complex sampling, Significance at $P < 0.05$

† Adjusted for age, BMI, sedentary time for study or leisure, sex, economic level, sleep time, subjective health status, subjective body shape image, smoking, and drinking alcohol histories.

‡ Adjusted for partial model plus dietary habit and exercise pattern

S2 Table Odd ratios of dietary habit or exercise pattern in 2020 compared to 2019 in high school student.

Dietary habit or PA	Number (%)		OR (95% CI)					
	2019	2020	Crude	P-value	Partial†	P-value	Full‡	P-value
Breakfast				<0.001*		0.182		0.276
0-1 time/week	7,079 (27.2)	7,238 (29.4)	1.00 (Ref)		1.00 (Ref)		1.00 (Ref)	
2-4 times/week	6,331 (24.3)	6,104 (24.8)	0.95 (0.90-1.00)		0.98 (0.93-1.04)		1.00 (0.95-1.05)	
≥ 5 times/week	12,597 (48.4)	11,257 (45.8)	0.88 (0.82-0.93)		0.95 (0.89-1.01)		0.96 (0.91-1.02)	
Fruit				<0.001*		0.003*		0.025*
0-2 times/week	11,809 (45.4)	11,961 (48.6)	1.00 (Ref)		1.00 (Ref)		1.00 (Ref)	
3-4 times/week	7,286 (28.0)	6,553 (26.6)	0.91 (0.86-0.95)		0.93 (0.89-0.98)		0.95 (0.90-1.00)	
≥ 5 times/week	6,912 (26.6)	6,085 (24.7)	0.89 (0.83-0.94)		0.92 (0.87-0.97)		0.93 (0.88-0.98)	
Soda drink				0.094		<0.001*		0.004*
0-2 times/week	16,473 (63.3)	15,664 (63.7)	1.00 (Ref)		1.00 (Ref)		1.00 (Ref)	
3-4 times/week	6,209 (23.9)	5,648 (23.0)	0.96 (0.90-1.01)		0.91 (0.87-0.96)		0.93 (0.88-0.97)	
≥ 5 times/week	3,325 (12.8)	3,287 (13.4)	1.01 (0.94-1.09)		0.93 (0.87-0.98)		0.92 (0.86-0.99)	
Sweet drink				<0.001*		<0.001*		<0.001*
0-2 times/week	12,437 (47.8)	12,400 (50.4)	1.00 (Ref)		1.00 (Ref)		1.00 (Ref)	
3-4 times/week	7,793 (30.3)	6,660 (27.1)	0.84 (0.80-0.88)		0.85 (0.81-0.88)		0.86 (0.83-0.90)	
≥ 5 times/week	5,777 (22.2)	5,539 (22.5)	0.95 (0.89-1.01)		0.94 (0.89-1.00)		0.98 (0.92-1.04)	
Fast food				0.007*		0.004*		<0.001*
0-2 times/week	19,364 (74.5)	18,045 (73.4)	1.00 (Ref)		1.00 (Ref)		1.00 (Ref)	
3-4 times/week	5,316 (20.4)	5,290 (21.5)	1.09 (1.03-1.15)		1.06 (1.01-1.12)		1.10 (1.05-1.17)	
≥ 5 times/week	1,327 (5.1)	1,264 (5.1)	1.02 (0.94-1.12)		0.91 (0.83-1.00)		0.95 (0.87-1.05)	
Vigorous PA				<0.001*		<0.001*		<0.001*

0 time/week	9,953 (38.3)	10,685 (43.4)	1.00 (Ref)		1.00 (Ref)		1.00 (Ref)	
1-2 times/week	9,344 (35.9)	7,838 (31.9)	0.76 (0.71-0.83)		0.78 (0.73-0.83)		0.76 (0.71-0.80)	
≥ 3 times/week	6,710 (25.8)	6,076 (24.7)	0.80 (0.72-0.90)		0.79 (0.73-0.84)		0.66 (0.61-0.71)	
Moderate PA				<0.001*		<0.001*		0.013*
0 time/week	10,034 (38.6)	10,351 (42.1)	1.00 (Ref)		1.00 (Ref)		1.00 (Ref)	
1-2 times/week	8,573 (33.0)	7,346 (29.9)	0.83 (0.78-0.88)		0.86 (0.81-0.90)		0.93 (0.88-0.97)	
≥ 3 times/week	7,400 (28.5)	6,902 (28.1)	0.87 (0.79-0.96)		0.88 (0.82-0.93)		0.96 (0.90-1.02)	
Strength Exercise				<0.001*		<0.001*		<0.001*
0 time/week	14,644 (56.3)	13,115 (53.3)	1.00 (Ref)		1.00 (Ref)		1.00 (Ref)	
1-2 times/week	5,864 (22.5)	5,443 (22.1)	1.01 (0.93-1.10)		1.06 (1.00-1.12)		1.20 (1.13-1.28)	
≥ 3 times/week	5,499 (21.1)	6,041 (24.6)	1.19 (1.06-1.33)		1.26 (1.18-1.34)		1.55 (1.45-1.67)	

* Multiple logistic regression analysis with complex sampling, Significance at $P < 0.05$

† Adjusted for age, BMI, sedentary time for study or leisure, sex, economic level, sleep time, subjective health status, subjective body shape image, smoking, and drinking alcohol histories.

‡ Adjusted for partial model plus dietary habit and exercise pattern

S3 Table Odd ratios of dietary habit or exercise pattern in 2020 compared to 2019 in men.

Dietary habit or PA	Number (%)		OR (95% CI)					
	2019	2020	Crude	P-value	Partial†	P-value	Full‡	P-value
Breakfast				<0.001*		0.006*		0.001*
0-1 time/week	7,343 (26.4)	7,335 (27.1)	1.00 (Ref)		1.00 (Ref)		1.00 (Ref)	
2-4 times/week	5,921 (21.3)	6,201 (22.9)	1.06 (1.01-1.12)		1.09 (1.03-1.15)		1.11 (1.05-1.17)	
≥ 5 times/week	14,512 (52.2)	13,497 (49.9)	0.94 (0.89-0.99)		1.02 (0.97-1.07)		1.03 (0.98-1.08)	
Fruit				<0.001*		0.027*		0.188
0-2 times/week	11,548 (41.6)	12,012 (44.4)	1.00 (Ref)		1.00 (Ref)		1.00 (Ref)	
3-4 times/week	7,681 (27.7)	7,227 (26.7)	0.91 (0.86-0.95)		0.94 (0.90-0.99)		0.96 (0.91-1.01)	
≥ 5 times/week	8,547 (30.8)	7,794 (28.8)	0.88 (0.83-0.94)		0.95 (0.90-1.00)		0.97 (0.92-1.02)	
Soda drink				<0.001*		<0.001*		<0.001*
0-2 times/week	15,437 (55.6)	15,616 (57.8)	1.00 (Ref)		1.00 (Ref)		1.00 (Ref)	
3-4 times/week	7,613 (27.4)	6,809 (25.2)	0.87 (0.84-0.91)		0.84 (0.80-0.87)		0.87 (0.83-0.91)	
≥ 5 times/week	4,726 (17.0)	4,608 (17.0)	0.94 (0.90-0.99)		0.87 (0.83-0.92)		0.89 (0.84-0.94)	
Sweet drink				<0.001*		<0.001*		<0.001*
0-2 times/week	13,060 (47.0)	13,536 (50.1)	1.00 (Ref)		1.00 (Ref)		1.00 (Ref)	
3-4 times/week	8,358 (30.1)	7,312 (27.0)	0.83 (0.79-0.86)		0.82 (0.79-0.86)		0.87 (0.83-0.91)	
≥ 5 times/week	6,358 (22.9)	6,185 (22.9)	0.94 (0.90-0.99)		0.91 (0.86-0.96)		0.98 (0.93-1.05)	
Fast food				0.592		0.009*		0.130
0-2 times/week	20,447 (73.6)	19,961 (73.8)	1.00 (Ref)		1.00 (Ref)		1.00 (Ref)	
3-4 times/week	5,709 (20.6)	5,549 (20.5)	1.00 (0.95-1.05)		0.97 (0.93-1.02)		1.03 (0.98-1.08)	
≥ 5 times/week	1,620 (5.8)	1,523 (5.6)	0.96 (0.89-1.04)		0.88 (0.82-0.96)		0.94 (0.86-1.02)	
Vigorous PA				<0.001*		<0.001*		<0.001*

0 time/week	5,010 (18.0)	7,137 (26.4)	1.00 (Ref)		1.00 (Ref)		1.00 (Ref)	
1-2 times/week	9,965 (35.9)	9,324 (35.4)	0.65 (0.61-0.69)		0.69 (0.65-0.73)		0.65 (0.62-0.70)	
≥ 3 times/week	12,801 (46.1)	10,572 (39.1)	0.57 (0.54-0.61)		0.62 (0.58-0.66)		0.51 (0.48-0.55)	
Moderate PA				<0.001*		<0.001*		0.116
0 time/week	6,823 (24.6)	8,051 (29.8)	1.00 (Ref)		1.00 (Ref)		1.00 (Ref)	
1-2 times/week	7,892 (28.4)	7,551 (27.9)	0.81 (0.77-0.85)		0.85 (0.81-0.89)		0.95 (0.90-1.00)	
≥ 3 times/week	13,061 (47.0)	11,431 (42.3)	0.73 (0.70-0.78)		0.80 (0.76-0.84)		0.95 (0.90-1.01)	
Strength Exercise				<0.001*		<0.001*		<0.001*
0 time/week	9,928 (35.7)	9,139 (33.8)	1.00 (Ref)		1.00 (Ref)		1.00 (Ref)	
1-2 times/week	8,496 (30.6)	7,618 (28.2)	0.95 (0.91-1.00)		1.02 (0.97-1.08)		1.20 (1.14-1.26)	
≥ 3 times/week	9,352 (33.7)	10,276 (38.0)	1.15 (1.09-1.21)		1.28 (1.21-1.35)		1.68 (1.59-1.78)	

* Multiple logistic regression analysis with complex sampling, Significance at $P < 0.05$

† Adjusted for age, BMI, sedentary time for study or leisure, sex, economic level, sleep time, subjective health status, subjective body shape image, smoking, and drinking alcohol histories.

‡ Adjusted for partial model plus dietary habit and exercise pattern

S4 Table Odd ratios of dietary habit or exercise pattern in 2020 compared to 2019 in women.

Dietary habit or PA	Number (%)		OR (95% CI)					
	2019	2020	Crude	P-value	Partial†	P-value	Full‡	P-value
Breakfast				<0.001*		0.194		0.110
0-1 time/week	7,112 (27.7)	7,451 (29.7)	1.00 (Ref)		1.00 (Ref)		1.00 (Ref)	
2-4 times/week	6,550 (25.5)	6,661 (26.5)	0.97 (0.93-1.02)		1.01 (0.96-1.07)		1.03 (0.98-1.09)	
≥ 5 times/week	12,023 (46.8)	10,994 (43.8)	0.88 (0.83-0.93)		0.97 (0.91-1.02)		0.98 (0.93-1.04)	
Fruit				<0.001*		<0.001*		<0.001*
0-2 times/week	10,516 (40.9)	11,322 (45.1)	1.00 (Ref)		1.00 (Ref)		1.00 (Ref)	
3-4 times/week	7,125 (27.7)	6,695 (26.7)	0.89 (0.85-0.94)		0.93 (0.89-0.98)		0.95 (0.90-0.99)	
≥ 5 times/week	8,044 (31.3)	7,089 (28.2)	0.83 (0.78-0.88)		0.87 (0.83-0.92)		0.88 (0.84-0.93)	
Soda drink				0.164		<0.001*		0.460
0-2 times/week	18,491 (53.5)	18,051 (58.5)	1.00 (Ref)		1.00 (Ref)		1.00 (Ref)	
3-4 times/week	4,920 (28.2)	4,673 (24.1)	0.98 (0.93-1.03)		0.91 (0.86-0.95)		0.97 (0.92-1.02)	
≥ 5 times/week	2,274 (18.3)	2,382 (17.4)	1.05 (0.98-1.13)		0.92 (0.86-0.99)		1.01 (0.94-1.09)	
Sweet drink				<0.001*		<0.001*		<0.001*
0-2 times/week	13,743 (53.5)	14,693 (58.5)	1.00 (Ref)		1.00 (Ref)		1.00 (Ref)	
3-4 times/week	7,249 (28.2)	6,038 (24.1)	0.76 (0.73-0.80)		0.75 (0.72-0.79)		0.76 (0.73-0.80)	
≥ 5 times/week	4,693 (18.3)	4,375 (17.4)	0.84 (0.80-0.89)		0.80 (0.75-0.84)		0.81 (0.76-0.87)	
Fast food				0.180		<0.001*		0.005*
0-2 times/week	19,789 (77.0)	19,302 (76.9)	1.00 (Ref)		1.00 (Ref)		1.00 (Ref)	
3-4 times/week	4,778 (18.6)	4,767 (19.0)	1.06 (0.96-1.16)		0.96 (0.91-1.01)		1.00 (0.95-1.06)	
≥ 5 times/week	1,118 (4.4)	1,037 (4.1)	1.09 (0.99-1.20)		0.79 (0.71-0.87)		0.85 (0.77-0.94)	
Vigorous PA				<0.001*		<0.001*		<0.001*

0 time/week	11,809 (46.0)	12,722 (50.7)	1.00 (Ref)		1.00 (Ref)		1.00 (Ref)	
1-2 times/week	9,158 (35.7)	8,087 (32.2)	0.81 (0.77-0.86)		0.83 (0.78-0.87)		0.79 (0.75-0.84)	
≥ 3 times/week	4,718 (18.4)	4,297 (17.1)	0.83 (0.77-0.89)		0.83 (0.80-0.89)		0.72 (0.67-0.78)	
Moderate PA				0.009*		0.080		0.154
0 time/week	11,824 (46.0)	11,957 (47.6)	1.00 (Ref)		1.00 (Ref)		1.00 (Ref)	
1-2 times/week	8,318 (32.4)	7,766 (30.9)	0.92 (0.88-0.97)		0.94 (0.89-0.99)		1.01 (0.96-1.06)	
≥ 3 times/week	5,543 (21.6)	5,383 (21.4)	0.95 (0.88-1.01)		0.96 (0.90-1.03)		1.07 (1.00-1.15)	
Strength Exercise				0.007*		0.002*		<0.001*
0 time/week	17,793 (693)	16,935 (67.5)	1.00 (Ref)		1.00 (Ref)		1.00 (Ref)	
1-2 times/week	5,416 (21.1)	5,506 (21.9)	1.07 (1.01-1.15)		1.09 (1.02-1.16)		1.18 (1.10-1.26)	
≥ 3 times/week	2,476 (9.6)	2,665 (10.6)	1.12 (1.04-1.21)		1.13 (1.05-1.22)		1.30 (1.19-1.42)	

* Multiple logistic regression analysis with complex sampling, Significance at $P < 0.05$

† Adjusted for age, BMI, sedentary time for study or leisure, sex, economic level, sleep time, subjective health status, subjective body shape image, smoking, and drinking alcohol histories.

‡ Adjusted for partial model plus dietary habit and exercise pattern
